# HD/FD and DF/AF with Fixed-Gain or Variable-Gain Protocol Switching Mechanism over Cooperative NOMA for Green-Wireless Networks

**DOI:** 10.3390/s19081845

**Published:** 2019-04-18

**Authors:** Thanh-Nam Tran, Miroslav Voznak

**Affiliations:** Faculty of Electrical Engineering and Computer Science, Technical University of Ostrava, 17. listopadu 2172/15, 708 00 Ostrava, Czech Republic; miroslav.voznak@vsb.cz

**Keywords:** protocol switching mechanism (PSM), half-duplex (HD), full-duplex (FD), decode-and-forward (DF), amplify-and-forward (AF), fixed gain (FG), variable gain (VG), cooperative NOMA, energy efficiency (EE), green-wireless networks (G-WNs)

## Abstract

This article studied the application of multiple protocol switching mechanism (PSM) over cooperating Non-Orthogonal Multiple Access (NOMA) networks to minimize the probability of outage and maximize the system throughput and energy efficiency (EE). This study investigated six scenarios: (1) a cooperative NOMA system with half-duplex (HD) and decode-and-forward (DF) protocols at the relay; (2) a cooperative NOMA system with full-duplex (FD) and DF protocols at the relay; (3) a cooperative NOMA system with HD and amplification amplify-and-forward (AF) with fixed-gain (FG) protocols at the relay; (4) a cooperative NOMA system with HD and amplification AF with variable-gain (VG) protocols at the relay; (5) a cooperative NOMA system with FD and amplification AF with FG protocols at the relay; (6) a cooperative NOMA system with FD and amplification AF with VG protocols at the relay. Based on the results of analysis and simulations, the study determined the transmission scenario for best system performance. This paper also proposed a mechanism to switch between HD/FD and DF/AF with FG/VG protocols in order to improve the quality of service (QoS) for users with a weak conditional channel. This mechanism can be deployed in future 5G wireless network sensors. Finally, EE was also assessed in relation to future green-wireless networks (G-WNs).

## 1. Introduction

Given the efficiency of superior spectral sharing and the possibility for a large number of connections at the same time slot/frequency [1,2], Non-Orthogonal Multi-Access technology (NOMA) in future wireless networks (5G) could serve a large user base. NOMA’s main technology is a superimposed signal sent to all users in a network by multiplexing the channel in the same power domain, but is different in terms of power factors [3]. At each receiving terminal, the end device, which has stronger conditional channel, is allocated a lower power coefficient than other devices and performs successive interference cancellation (SIC) by treating other users’ information as interference before detecting its own information [4]. The user, which has the weakest channel condition, only has to decode its own information by applying SIC.

Some initial studies have contributed significantly to the implementation of NOMA in the future. A complete survey in the field of NOMA includes early introduction, recent technologies and future research trends, especially discussions about NOMA’s outstanding advantages over previous technologies [5]. The authors analyzed the system performance based on resource allocation [6,7]. In [6], the algorithm distributes power to users in different clusters to balance the system throughput and QoS fairness. The authors ensure fairness for users based on a reasonable allocation of power. The allocation coefficients can be allocated by the user’s channel status information (CSI) [8,9]. In another study, the authors investigated the system performance with the assumption of imperfect channel state information (CSI) over the amplify-and-forward (AF) protocol [10]. However, the authors also assumed that only a single antenna has been installed at each node. The impact of the loop interference (LI) channel generated when a relay is equipped with a twin antenna and operated in the FD protocol has therefore not been analyzed. It is a motivation for us to investigate the impact of the LI channel in the AF protocol.

Recently, relaying technology has raised much research interest as an effective solution to the fading resistance. In the cooperative NOMA model, a user with the strongest channel condition is selected as a receiving device and forwards the superimposed signals to users with weaker channel conditions. Therefore, the scope/distance of the network is expanded and the reliability of the network is enhanced by improving QoS for users [11,12,13,14,15]. In [16], the authors investigated the outage performance of the AF and decode-and-forward (DF) relaying schemes. The authors also proposed using a full-duplex (FD) protocol instead of the half-duplex (HD) protocol to avoid wasting time slots [17]. Although a cooperative NOMA network improves QoS for remote users, it also increases bandwidth costs. This problem can be solved by applying the FD relay technique. The FD relay receives and forwards a signal simultaneously in the same frequency band [18]. A disadvantage of FD relaying is the impact of the loop interference channel from its own transmitter antenna modeled as a fading channel. Loop interference channels are the main challenge in implementing FD relays [19]. The authors proposed interference cancellation techniques, including passive cancellation, active analog cancellation, and active digital cancellation [20]. Studies [21] and [22] discussed two main types of FD relay techniques, namely FD AF relaying and FD DF relaying. The authors also investigated a cognitive radio NOMA in FD/HD relay [23]. The mechanism of random switching between HD/FD relays is on transmit power adaptation [24]. Another full study on HD/FD relay DF protocol is evaluated in [25]. Taking up on previous research results, the question whether HD or FD protocol is more suitable arises. A disadvantage of FD protocol is that it is affected by the LI channel, while HD protocol does not have any LI channel. The FD protocol, however, has a better frequency efficiency than the HD protocol. This study proposes a protocol switching mechanism to effectively use the advantages of each protocol. This mechanism can be deployed as a sensor for relaying in future wireless networks. The authors also investigated the HD/FD relay and AF protocol with a fixed gain (FG) [26]. Through the results, the authors demonstrated that the NOMA system outperforms compared to orthogonal multiple access (OMA) system over the Nakagami-*m* fading channels. AF with a variable gain (VG) is less interesting in research because of its complexity. Last, this paper also investigated HD/FD relays not only using the AF protocol with FG but also AF with VG.

Certain studies have made significant contributions in the field of cooperative NOMA. Research results have shown that system performance can be improved by selecting the appropriate relay. Ding et al. [27] proposed a two-stage relay selection strategy that outperforms max-min relay selection. Another potential technology in the future 5G network is radio frequency energy harvesting (EH) [28]. However, the initial studies on high-power wireless power transmission show that high-power devices are potentially dangerous to health, thus inhibiting further development of wireless EH. A complete survey of the advantages of simultaneous wireless information and power transfer (SWIPT) over other wireless power transfer (WPT) techniques is in [29]. In [30], the authors surveyed most SWIPT technologies, including SWIPT enabled multi-carrier systems, full-duplex SWIPT systems, etc. Given the explosion in the number of networked devices, e.g., Internet of things (IoTs) devices, the energy issue is particularly important when it comes to research and implementation in G-WNs . A solution for simultaneous data and energy transmission is proposed in [31]. Although the wireless EH solution has not achieved practical effectiveness yet, this study suggests a solution for energy savings that can be easily deployed in applications rather than wireless EH, based on capacity surveys for the best EE of the system.

The main contributions of this study are:

- An investigation into the system performance of cooperative NOMA under six scenarios: (1) HD and DF relay; (2) FD and DF relay; (3) HD and AF with FG relay; (4) FD and AF with FG relay; (5) HD and AF with VG relay; (6) FD and AF with VG relay. The outage probability of each scenario is presented in a closed form.
- A proposal of a mechanism for switching protocols and optimizing system performance by selecting the best protocol to forward a signal to the next user.
- An investigation into the system performance on different signal-to-noise-ratios (SNRs) to find a suitable means of transmitting power to avoid wasting energy. Energy saving is required in G-WNs.
- The results coming from the analysis and simulation of outage probability, system throughput and EE are performed by Matlab (This paper used Matlab software version R2017b, made by The MathWorks, Inc., 3 Apple Hill Drive Natick, MA 01760 USA 508-647-7000) software. In addition, an algorithm used for Monte Carlo simulation is also proposed for investigating the outage probability of individual scenarios. The simulation results are used for verifying the analysis results. The figures are presented clearly and accurately in order to demonstrate our propositions.

The article is structured as follows: first, an experimental model is proposed. Next, six different scenarios are analyzed. The third section analyzes system performance on outage probability, system throughput and energy efficiency in all six proposed scenarios. In the fourth section, numerical results are presented and the figures are clearly and accurately discussed. A summary of the study is presented in the conclusions.

## 2. Experimental Models

**Figure 1:**
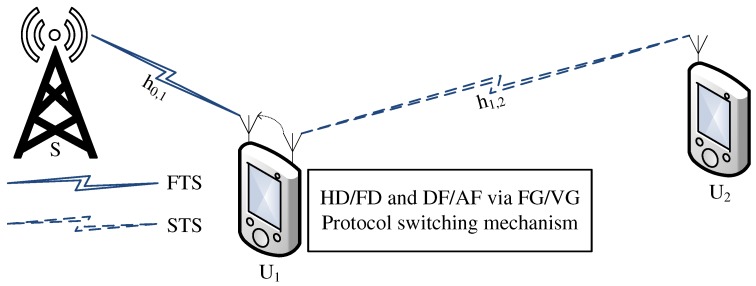
HD/FD and DF/AF with FG/VG relay over cooperative NOMA system.

In the system model (Figure 1), two users are waiting to receive the signals with the assumption that the user $U_{1}$’s channel is in a better condition than that of user $U_{2}$. Both users are over Rayleigh fading channels. As $U_{2}$ has poor channel conditions, it, instead of receiving the down-link signals directly from the base station, requires a support of a relay for the relaying signals. This paper assumes that $U_{1}$ can be used as a cooperative relay. Another assumption is that $U_{1}$ can work in all six protocols: HD and DF, FD and DF, HD and AF with FG, FD and AF with FG, HD and AF with VG, FD and AF with VG. This paper analyzes all six protocols to find out the best protocol. Based on these facilities, the study proposes using a wireless sensor to switch between and select protocols to optimize the system performance.

In Figure 1, although the FD protocol can send and receive data simultaneously, an initial mixed signal is sent from the BS to the relay in the first time slot. The relay decodes the $x_{2}$ symbol and removes $x_{2}$ from the mixed signal before decoding its own $x_{1}$ symbol. The information in the $x_{2}$ symbol is then restored and forwarded to $U_{2}$ in the second time slot. However, the forwarded signal from the transmitter antenna generates a loop channel to the receiver antenna while $U_{2}$ receives another signal from the BS. Thus, the cooperative NOMA system requires two time slots to transmit a superposed signal from the BS to the second user $U_{2}$.

### 2.1. First Time Slot (FTS)

According to the NOMA theory, the BS sends a superimposed signal to $U_{1}$ in the FTS expressed by

(1) $$S=\left(\sqrt{\alpha_{1}P_{0}}x_{1}+\sqrt{\alpha_{2}P_{0}}x_{2}\right),$$

where $\alpha_{1}<\alpha_{2}$ and $\alpha_{1}+\alpha_{2}=1$ are in accordance with condition $\left|h_{0,1}\right|>\left|h_{0,2}\right|$, and $P_{0}$ the transmission power of the BS and $x_{i}$ for $i=\left\{1,2\right\}$ is the information symbol of each user, sequentially.

Therefore, the received signal at $U_{1}$ can be expressed as:

(2) $$y_{1}^{\Omega }=h_{0,1}\sqrt{P_{0}}\sum_{k=1}^{2}\sqrt{\alpha_{k}}x_{k}+\varepsilon h_{1,1}\sqrt{P_{1}}\tilde{x}+n_{1},$$

where $h_{0,1}$ is denoted as the transmission channel from BS to $U_{1}$, $h_{1,1}$ is the LI channel from transmitter antenna to receiver one at $U_{1}$ and $n_{1}$ is the additive white Gaussian noise (AWGN) at $U_{1}$ for $n_{1}∼CN\left(0,N_{0}\right)$ with zero mean and variance $N_{0}$. $\Omega =\left\{HD,FD\right\}$ is the HD/FD switching mode with ϵ state factor. If ϵ=0, the relay operates in the HD mode. If ϵ=1, the relay operates in the FD mode.

$U_{1}$ needs two phases to decode its own information symbol. In the first phase, $U_{1}$ decodes the $x_{2}$ symbol by dealing with the $x_{1}$ symbol, the LI channel $h_{1,1}$ and AWGN $n_{1}$. The signal-to-interference-plus-noise ratio (SINR) can then be expressed as:

(3) $$\gamma_{1\to 2}^{\Omega }=\frac{{\left|h_{0,1}\right|}^{2}\alpha_{2}P_{0}}{{\left|h_{0,1}\right|}^{2}\alpha_{1}P_{0}+\varepsilon {\left|h_{1,1}\right|}^{2}P_{1}+N_{0}}=\frac{{\left|h_{0,1}\right|}^{2}\alpha_{2}\rho_{0}}{{\left|h_{0,1}\right|}^{2}\alpha_{1}\rho_{0}+\varepsilon {\left|h_{1,1}\right|}^{2}\rho_{1}+1},$$

where $\rho_{0}=P_{0}/N_{0}$.

In the second phase, after $U_{1}$ has decoded the $x_{2}$ symbol, $x_{2}$ would be removed from the superimposed signal as noise. $U_{1}$ decodes its own symbol $x_{1}$ after removing $x_{2}$ by dealing with AWGN $n_{1}$ and the LI channel $h_{1,1}$. SINR can then be expressed as:

(4) $$\gamma_{1\to 1}^{\Omega }=\frac{{\left|h_{0,1}\right|}^{2}\alpha_{1}P_{0}}{\varepsilon {\left|h_{1,1}\right|}^{2}P_{1}+N_{0}}=\frac{{\left|h_{0,1}\right|}^{2}\alpha_{1}\rho_{0}}{\varepsilon {\left|h_{1,1}\right|}^{2}\rho_{1}+1}.$$

The instantaneous achievable bit rate of $U_{1}$ when $U_{1}$ decodes the $x_{j}$ symbol can therefore be expressed by:

(5) $$R_{1\to j}^{\Omega }=\frac{\log_{2}\left(1+\gamma_{1\to j}^{\Omega }\right)}{2},$$

where $j=\left\{2,1\right\}$.

### 2.2. Second Time Slot (STS)

In STS, $U_{1}$ will forward a mixed signal to $U_{2}$ using either the DF protocol or the AF protocol with FG or VG by the PSS.

#### 2.2.1. DF Protocols at the Relay

Once the $x_{2}$ symbol has been decoded and removed from the superimposed signal, $x_{2}$ is restored and sent to $U_{2}$. Therefore, $U_{2}$ will receive a signal expressed as:

(6) $$y_{2}^{DF}=h_{1,2}\sqrt{P_{1}}x_{2}+n_{2},$$

where $h_{1,2}$ is the transmission channel from $U_{1}$ to $U_{2}$, $P_{1}$ is the transmission power of $U_{1}$ and $n_{2}$ is the AWGN of $U_{2}$.

$U_{2}$ decodes its own $x_{2}$ symbol by removing AWGN $n_{2}$ from the received signal. Meanwhile, SINR can be expressed by:

(7) $$\gamma_{2\to 2}^{DF}=\frac{{\left|h_{1,2}\right|}^{2}P_{1}}{N_{0}}={\left|h_{1,2}\right|}^{2}\rho_{1}.$$

The instantaneous achievable bit rate of $U_{2}$ in the DF protocol is expressed as:

(8) $$R_{2\to 2}^{DF}=\frac{\log_{2}\left(1+\gamma_{2\to 2}^{DF}\right)}{2}.$$

#### 2.2.2. AF with FG/VG Protocols at the Relay

Where the $U_{1}$ relay uses the AF protocol, $U_{1}$ will amplify the received signal by the amplification factor $\kappa_{\omega }$, for $\omega =\left\{FG,VG\right\}$, before forwarding the superimposed signal to $U_{2}$.

The $\kappa_{\omega }$ amplification coefficient for FG and VG, respectively, are given as follows:

(9a) $$\begin{array}{cc} \kappa_{\omega } & \overset{\Delta }{\;=}\sqrt{\frac{P_{1}}{P_{0}E\left[{\left|h_{1,2}\right|}^{2}\right]+N_{0}}}=\sqrt{\frac{\rho_{1}}{\rho_{0}\sigma_{1,2}^{2}+1}}, \end{array}$$

(9b) $$\begin{array}{c} \overset{\wedge }{\;=}\sqrt{\frac{P_{1}}{P_{0}{\left|h_{1,2}\right|}^{2}+N_{0}}}=\sqrt{\frac{\rho_{1}}{\rho_{0}{\left|h_{1,2}\right|}^{2}+1}}, \end{array}$$

where $\omega \overset{\Delta }{\;=}FG$ or $\omega \overset{\wedge }{\;=}VG$.

Therefore, the received signal at $U_{2}$ is expressed as:

(10) $$y_{2}^{\Omega ,\omega }=\kappa_{\omega }h_{1,2}\sqrt{P_{1}}y_{1}^{\Omega }+n_{2},$$

where $\kappa_{\omega }$ is given by (9a) or (9b) and $y_{1}^{\Omega }$ is given by (2).

By submitting (2) and (9a) or (9b) into (10), $U_{2}$ decodes its own $x_{2}$ symbol by removing the $x_{1}$ symbol, removing the LI channel if $U_{1}$ works in Ω=FD mode, removing AWGN $n_{1}$ of $U_{1}$ and removing its own AWGN $n_{2}$. Therefore, SINR can be expressed as follows:

(11) $$\gamma_{2\to 2}^{\Omega ,\omega }=\frac{{\left|h_{0,1}\right|}^{2}\alpha_{2}\rho_{0}}{{\left|h_{0,1}\right|}^{2}\alpha_{1}\rho_{0}+\varepsilon {\left|h_{1,1}\right|}^{2}\rho_{1}+1+\psi_{\omega }},$$

where $\psi_{\omega }$ is given by:

(12) $$\psi_{\omega }=\frac{1}{\kappa_{\omega }^{2}{\left|h_{1,2}\right|}^{2}\rho_{1}},$$

for $\omega \overset{\Delta }{\;=}FG$ or $\omega \overset{\wedge }{\;=}VG$.

As with (8), the instantaneous bit rate threshold of $U_{2}$ in AF protocols with FG/VG can be rewritten as:

(13) $$R_{2\to 2}^{\Omega ,\omega }=\frac{\log_{2}\left(1+\gamma_{2\to 2}^{\Omega ,\omega }\right)}{2}.$$

## 3. System Performance Analysis

Previous research results showed the feasibility of deploying a cooperative relay with HD/FD and DF protocols to resist fading. A mixed signal was transmitted through the network with the support of the N−1 HD/FD relay before reaching the *N*-th user [31]. However, the AF protocol is less studied than the DF protocol because of its complexity in SIC. By contrast, the authors had a full study of the AF protocol with FG [26]. Even so, it lacks a comparison to the AF protocol with VG. These research results were our motivation to seek a complete analysis and evaluation of the advantages of each protocol.

In this section, we analyze outage probability, system throughput and EE to evaluate the system performance of a cooperative NOMA system in six proposed scenarios: (1) HD and DF protocols at the relay; (2) FD and DF protocols at the relay; (3) HD and AF with FG protocols at the relay; (4) FD and AF with FG protocols at the relay; (5) HD and AF with VG protocols at the relay; (6) FD and AF with VG protocols at the relay, respectively. The article then proposes a mechanism for switching protocols to optimize the system performance.

### 3.1. Outage Probability

The probability density function (PDF) and cumulative distribution function (CDF) of the Rayleigh fading channel can be expressed as follows, respectively:

(14) $$f_{{\left|h_{a,b}\right|}^{2}}\left(x\right)=\frac{1}{\sigma_{a,b}^{2}}e^{-\frac{x}{\sigma_{a,b}^{2}}},$$

and

(15) $$F_{{\left|h_{a,b}\right|}^{2}}\left(x\right)=1-e^{-\frac{x}{\sigma_{a,b}^{2}}},$$

where random independent variable x≥0

**Theorem** **1.** *The outage of signal transmission of $U_{1}$ will occur when $U_{1}$ cannot successfully decode either $x_{1}$ or $x_{2}$ symbol. Specifically, this outage will occur when there is one of the following cases:*

- ***Case 1*** : The instantaneous bit rate $R_{1\to 2}^{\Omega }$ cannot reach to the bit rate threshold $R_{2}^{*}$, in other words $R_{1\to 2}^{\Omega }<R_{2}^{*}$.
- ***Case 2*** : The instantaneous bit rate $R_{1\to 2}^{\Omega }$ can reach to the bit rate threshold $R_{2}^{*}$ but the instantaneous bit rate $R_{1\to 1}^{\Omega }$ cannot reach to the bit rate threshold $R_{1}^{*}$, in other words $R_{1\to 2}^{\Omega }>R_{2}^{*}$, and $R_{1\to 1}^{\Omega }<R_{1}^{*}$.

*Ultimately, the outage probability of $U_{1}$ can be expressed as:*

(16) $${\Theta_{1}}^{\Omega }=1-\prod_{j=1}^{2}\mathrm{Pr}\left(R_{1\to j}^{\Omega }>R_{j}^{*}\right)=1-\mathrm{Pr}\left(R_{1\to 2}^{\Omega }>R_{2}^{*},R_{1\to 1}^{\Omega }>R_{1}^{*}\right),$$

where $R_{j}^{*}$ is the minimum bit rate threshold of $U_{j}$ that needs to be achieved.

The expression (16) can be solved and represented in a closed form as:

(17) $$\Theta_{1}^{\Omega }=1-e^{-\left(\frac{R_{1}^{**}}{\alpha_{1}\rho_{0}\sigma_{0,1}^{2}}\right)}\frac{\alpha_{1}\rho_{0}\sigma_{0,1}^{2}}{\alpha_{1}\rho_{0}\sigma_{0,1}^{2}+\varepsilon R_{1}^{**}\rho_{1}\sigma_{1,1}^{2}}\underset{\lambda_{1}}{\underset{︸}{e^{-\frac{R_{2}^{**}}{\left(\alpha_{2}-\alpha_{1}R_{2}^{**}\right)\rho_{0}\sigma_{0,1}^{2}}}\frac{\left(\alpha_{2}-\alpha_{1}R_{2}^{**}\right)\rho_{0}\sigma_{0,1}^{2}}{\left(\alpha_{2}-\alpha_{1}R_{2}^{**}\right)\rho_{0}\sigma_{0,1}^{2}+\varepsilon R_{2}^{**}\rho_{1}\sigma_{1,1}^{2}}}},$$

where $R_{i}^{**}=2^{2R_{i}^{*}}-1$ for $i=\left\{1,2\right\}$. Where Ω=HD or Ω=FD for ε=0 or ε=1, then it is paired.

For the proof of Theorem 1, see the Appendix A.

**Theorem** **2.** *The outage of signal transmission of $U_{2}$ will occur when either $U_{1}$ or $U_{2}$ cannot successfully decode $x_{2}$ symbol. Specifically, this outage will occur when there is one of the following cases:*

- ***Case 1*** : The instantaneous bit rate $R_{1\to 2}^{\Omega }$ cannot reach the bit rate threshold $R_{2}^{*}$, in other words $R_{1\to 2}^{\Omega }<R_{2}^{*}$.
- ***Case 2*** : The instantaneous bit rate $R_{1\to 2}^{\Omega }$ can reach to the bit rate threshold $R_{2}^{*}$ but the instantaneous bit rate $R_{2\to 2}^{\Omega }$ cannot reach to the bit rate threshold $R_{2}^{*}$, in other words, $R_{1\to 2}^{\Omega }>R_{2}^{*}$, and $R_{2\to 2}^{\Omega }<R_{2}^{*}$.

*Ultimately, the outage probability of $U_{2}$ can be expressed as:*

(18) $${\Theta_{2}}^{\Omega ,\omega }=1-\prod_{i=1}^{2}\mathrm{Pr}\left(R_{i\to 2}^{\Omega ,\omega }>R_{2}^{*}\right)=\mathrm{Pr}\left(R_{1\to 2}^{\Omega }<R_{2}^{*}\right)+\mathrm{Pr}\left(R_{1\to 2}^{\Omega }>R_{2}^{*},R_{2\to 2}^{\Omega ,\omega }<R_{2}^{*}\right).$$

#### 3.1.1. HD and DF Protocols at the Relay (Ω=HD,ω=DF)

**Remark** **1.** *In this scenario, $U_{1}$ is operated by HD and DF protocols. Therefore, Theorem 2 shown as (18) can be rewritten as well as solved in closed form as:*

(19) $$\begin{array}{cc} {\Theta_{2}}^{HD,DF} & =\prod_{i=1}^{2}\mathrm{Pr}\left(R_{i\to 2}^{HD,DF}<R_{2}^{*}\right)=\mathrm{Pr}\left(R_{1\to 2}^{HD}<R_{2}^{*}\right)+\mathrm{Pr}\left(R_{1\to 2}^{HD}>R_{2}^{*},R_{2\to 2}^{HD,DF}<R_{2}^{*}\right)\\ & =\left(1-\lambda_{1}\right)+\lambda_{1}\left(1-\underset{\lambda_{2}}{\underset{︸}{e^{-\frac{R_{2}^{**}}{\rho_{1}\sigma_{1,2}^{2}}}}}\right)=1-\lambda_{1}\lambda_{2}, \end{array}$$

*where $\lambda_{1}$ is given by (17) for ε=0.*

*For the proof of Remark 1, see the Appendix A.*

#### 3.1.2. FD and DF Protocols at the Relay (Ω=FD,ω=DF)

**Remark** **2.** *In this scenario, $U_{1}$ is operated by FD and DF protocols. Therefore, the Theorem 2 shown as (18) can be rewritten as well as solved and in closed form as:*

(20) $$\begin{array}{cc} {\Theta_{2}}^{FD,DF} & =\prod_{i=1}^{2}\mathrm{Pr}\left(R_{i\to 2}^{FD,DF}<R_{2}^{*}\right)=\mathrm{Pr}\left(R_{1\to 2}^{FD}<R_{2}^{*}\right)+\mathrm{Pr}\left(R_{1\to 2}^{FD}>R_{2}^{*},R_{2\to 2}^{FD,DF}<R_{2}^{*}\right)\\ & =\left(1-\lambda_{1}\right)+\left(\lambda_{1}\left(1-\lambda_{2}\right)\right)=1-\lambda_{1}\lambda_{2}, \end{array}$$

*where $\lambda_{1}$ and $\lambda_{2}$ are given by (17) and (19) for ε=1, respectively. If ε in (20) equals zero, (20) becomes (19).*

*For the proof of Remark 2, see the Appendix A.*

#### 3.1.3. HD and AF with FG Protocols at the Relay ($\Omega =HD,\omega \overset{\Delta }{\;=}FG$)

**Remark** **3.** *In this scenario, $U_{1}$ is operated by HD and AF with FG protocols. Before forwarding a signal to $U_{2}$, $U_{1}$ amplifies the received signal shown as (2), where ε=0, by the amplification coefficient $\kappa_{FG}$ given by (9a). The outage probability of $U_{2}$ is then expressed in closed form as:*

(21) $$\begin{array}{cc} {\Theta_{2}}^{HD,FG} & =1-\prod_{i=1}^{2}\mathrm{Pr}\left(R_{i\to 2}^{HD,FG}>R_{2}^{*}\right)=\mathrm{Pr}\left(R_{1\to 2}^{HD}<R_{2}^{*}\right)+\mathrm{Pr}\left(R_{1\to 2}^{HD}>R_{2}^{*},R_{2\to 2}^{HD,FG}<R_{2}^{*}\right)\\ & =\left(1-\lambda_{1}\right)+\lambda_{1}\left(1-\frac{2e^{-\frac{R_{2}^{**}}{\left(\alpha_{2}-\alpha_{1}R_{2}^{**}\right)\rho_{0}\sigma_{0,1}^{2}}}\sqrt{\frac{1}{\sigma_{1,2}^{2}}}K_{1}\left(\frac{2\sqrt{\frac{1}{\sigma_{1,2}^{2}}}}{\sqrt{\frac{\left(\alpha_{2}-\alpha_{1}R_{2}^{**}\right)\rho_{0}\rho_{1}^{2}\sigma_{0,1}^{2}}{R_{2}^{**}\left(1+\rho_{0}\sigma_{1,2}^{2}\right)}}}\right)}{\sqrt{\frac{\left(\alpha_{2}-\alpha_{1}R_{2}^{**}\right)\rho_{0}\rho_{1}^{2}\sigma_{0,1}^{2}}{R_{2}^{**}\left(1+\rho_{0}\sigma_{1,2}^{2}\right)}}}\right), \end{array}$$

*where $\lambda_{1}$ is given by (17) for ε=0 and $K_{n}\left(.\right)$ is denoted as a modified BesselK function.*

*For the proof of Remark 3, see the Appendix A.*

#### 3.1.4. FD and AF with FG Protocols at the Relay ($\Omega =FD,\omega \overset{\Delta }{\;=}FG$)

**Remark** **4.** *In this scenario, $U_{1}$ is operated by FD and AF with FG protocols. Before forwarding a signal to $U_{2}$, $U_{1}$ amplifies the received signal shown as (2), where ε=1, by the amplification coefficient $\kappa_{FG}$ given by (9a). The outage probability of $U_{2}$ is then expressed in closed form as:*

(22) $$\begin{array}{c} {\Theta_{2}}^{FD,FG}=1-\prod_{i=1}^{2}\mathrm{Pr}\left(R_{i\to 2}^{FD,FG}>R_{2}^{*}\right)=\mathrm{Pr}\left(R_{1\to 2}^{FD}<R_{2}^{*}\right)+\mathrm{Pr}\left(R_{1\to 2}^{FD}>R_{2}^{*},R_{2\to 2}^{FD,FG}<R_{2}^{*}\right)\\ =\left(1-\lambda_{1}\right)+\lambda_{1}\left(1-\frac{2e^{-\frac{R_{2}^{**}}{\left(\alpha_{2}-\alpha_{1}R_{2}^{**}\right)\rho_{0}\sigma_{0,1}^{2}}}\sqrt{\frac{1}{\sigma_{1,2}^{2}}}\left(\alpha_{2}-\alpha_{1}R_{2}^{**}\right)\rho_{0}\sigma_{0,1}^{2}K_{1}\left(\frac{2\sqrt{\frac{1}{\sigma_{1,2}^{2}}}}{\sqrt{\frac{\left(\alpha_{2}-\alpha_{1}R_{2}^{**}\right)\rho_{0}\rho_{1}^{2}\sigma_{0,1}^{2}}{R_{2}^{**}\left(1+\rho_{0}\sigma_{1,2}^{2}\right)}}}\right)}{\left(\left(\alpha_{2}-\alpha_{1}R_{2}^{**}\right)\rho_{0}\sigma_{0,1}^{2}+\varepsilon R_{2}^{**}\rho_{1}\sigma_{1,1}^{2}\right)\sqrt{\frac{\left(\alpha_{2}-\alpha_{1}R_{2}^{**}\right)\rho_{0}\rho_{1}^{2}\sigma_{0,1}^{2}}{R_{2}^{**}\left(1+\rho_{0}\sigma_{1,2}^{2}\right)}}}\right). \end{array}$$

*where ε in (22) equals zero, (22) becomes (21).*

*For the proof of Remark 4, see the Appendix A.*

#### 3.1.5. HD and AF with VG Protocols at the Relay ($\Omega =HD,\omega \overset{\wedge }{\;=}VG$)

**Remark** **5.** *In this scenario, $U_{1}$ is operated by HD and AF with VG protocols. Before forwarding a signal to $U_{2}$, $U_{1}$ amplifies the received signal shown as (2), where ε=0, by the amplification coefficient $\kappa_{VG}$ given by (9b). The outage probability of $U_{2}$ is then expressed in closed form as:*

(23) $$\begin{array}{cc} {\Theta_{2}}^{HD,VG} & =1-\prod_{i=1}^{2}\mathrm{Pr}\left(R_{i\to 2}^{HD,VG}>R_{2}^{*}\right)=\mathrm{Pr}\left(R_{1\to 2}^{HD}<R_{2}^{*}\right)+\mathrm{Pr}\left(R_{1\to 2}^{HD}>R_{2}^{*},R_{2\to 2}^{HD,VG}<R_{2}^{*}\right)\\ & =\left(1-\lambda_{1}\right)+\lambda_{1}\left(1-\frac{2e^{\frac{R_{2}^{**}\left(\rho_{1}^{2}+\rho_{0}\right)}{\left(\alpha_{1}R_{2}^{**}-\alpha_{2}\right)\rho_{0}\rho_{1}^{2}\sigma_{0,1}^{2}}}\sqrt{\frac{1}{\sigma_{1,2}^{2}}}K_{1}\left(\frac{2\sqrt{\frac{1}{\sigma_{1,2}^{2}}}}{\sqrt{\frac{\left(\alpha_{1}R_{2}^{**}-\alpha_{2}\right)\rho_{0}\rho_{1}^{2}\sigma_{0,1}^{2}}{R_{2}^{**}}}}\right)}{\sqrt{\frac{\left(\alpha_{1}R_{2}^{**}-\alpha_{2}\right)\rho_{0}\rho_{1}^{2}\sigma_{0,1}^{2}}{R_{2}^{**}}}}\right). \end{array}$$

*For the proof of Remark 5, see the Appendix A.*

#### 3.1.6. FD and AF with VG Protocols at the Relay ($\Omega =FD,\omega \overset{\Delta }{\;=}VG$)

**Remark** **6.** *In this scenario, $U_{1}$ is operated by FD and AF with VG protocols. Before forwarding a signal to $U_{2}$, $U_{1}$ amplifies the received signal shown as (2), where ε=1, by the amplification coefficient $\kappa_{VG}$ given by (9b). The outage probability of $U_{2}$ is then expressed in closed form as:*

(24) $$\begin{array}{c} {\Theta_{2}}^{FD,VG}=1-\prod_{i=1}^{2}\mathrm{Pr}\left(R_{i\to 2}^{\Omega ,\omega }>R_{2}^{*}\right)=\mathrm{Pr}\left(R_{1\to 2}^{FD}<R_{2}^{*}\right)+\mathrm{Pr}\left(R_{1\to 2}^{FD}>R_{2}^{*},R_{2\to 2}^{FD,VG}<R_{2}^{*}\right)\\ =\left(1-\lambda_{1}\right)+\lambda_{1}\left(1-\frac{2e^{-\frac{R_{2}^{**}\left(\rho_{1}^{2}+\rho_{0}\right)}{\left(\alpha_{2}-\alpha_{1}R_{2}^{**}\right)\rho_{0}\rho_{1}^{2}\sigma_{0,1}^{2}}}\sqrt{\frac{1}{\sigma_{1,2}^{2}}}\left(\alpha_{2}-\alpha_{1}R_{2}^{**}\right)\rho_{0}\sigma_{0,1}^{2}K_{1}\left(\frac{2\sqrt{\frac{1}{\sigma_{1,2}^{2}}}}{\sqrt{\frac{\left(\alpha_{2}-\alpha_{1}R_{2}^{**}\right)\rho_{0}\rho_{1}^{2}\sigma_{0,1}^{2}}{R_{2}^{**}}}}\right)}{\left(\left(\alpha_{2}-\alpha_{1}R_{2}^{**}\right)\rho_{0}\sigma_{0,1}^{2}+\varepsilon R_{2}^{**}\rho_{1}\sigma_{1,1}^{2}\right)\sqrt{\frac{\left(\alpha_{2}-\alpha_{1}R_{2}^{**}\right)\rho_{0}\rho_{1}^{2}\sigma_{0,1}^{2}}{R_{2}^{**}}}}\right). \end{array}$$

*where ε in (24) equals zero, (24) becomes (23).*

*For the proof of Remark 6, see the Appendix A.*

### 3.2. System Throughput

The sum of achievable received data at $U_{i}$, which is also referred to as the system throughput $P_{sys}^{\Omega ,\omega }$, is the sum of the throughput results of all $U_{i}$ in the system, expressed as:

(25) $$\begin{array}{c} P_{sys}^{\Omega ,\omega }=P_{1}^{\Omega }+P_{2}^{\Omega ,\omega }=\left(1-\Theta_{1}^{\Omega }\right)R_{1}^{*}+\left(1-\Theta_{2}^{\Omega ,\omega }\right)R_{2}^{*}, \end{array}$$

where $\Omega =\left\{HD,FD\right\}$ and $\omega =\left\{DF,FG,VG\right\}$.

### 3.3. Energy Efficiency

Technological development has significantly increased the amount of electricity consumed and seriously affected the living environment. Thus, the minimal energy consumption on every bit of data transmitted through the network is an essential requirement of next generation mobile networks. In this section, the paper evaluates the EE of each scenario:

(26) $$EE_{sys}^{\Omega ,\omega }=\frac{P_{1}^{\Omega }+P_{2}^{\Omega ,\omega }}{\rho_{0}+\rho_{1}}=\frac{\left(1-\Theta_{1}^{\Omega }\right)R_{1}^{*}+\left(1-\Theta_{2}^{\Omega ,\omega }\right)R_{2}^{*}}{\rho_{0}+\rho_{1}}.$$

### 3.4. Protocol Switching Mechanism

In this section, the study proposes implementing a protocol switching mechanism. Of the six protocols analyzed above, none surpasses any of the other protocols significantly. Each protocol has its own advantages in different situations. The results of the analysis presented in the next section will demonstrate the advantages of each protocol more clearly. Therefore, it is necessary that the relay needs to be equipped with a sensor that can switch between the six protocols in order to optimize the system performance.

**Figure 2:**
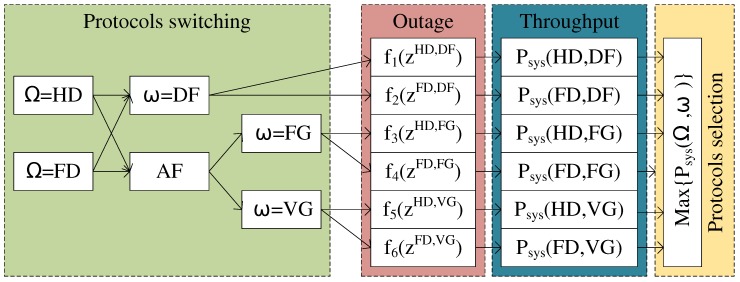
Protocol switching mechanism.

Figure 2 shows the mechanism of switching between protocols HD/FD and DF/AF with FG/VG. Each protocol is submitted into its corresponding analysis function. The results of the analysis are used to decide which protocol is optimal and should be applied to forward a signal to the next user at the moment of evaluation.

**Proposition** **1.** *Investigation of the outage probability in all six scenarios by the relevant functions to select the best protocol with the lowest outage probability result in order to optimize QoS for the network users.*

The minimum outage probability results in HD and DF/AF with FG/VG protocols is selected by:

(27) $$PSS_{\Theta }^{HD,\omega }=\min\left\{\Theta_{1}^{HD}\right\}+\min\left\{\Theta_{2}^{HD,\omega }\right\},$$

and the minimum outage probability results in FD and DF/AF with FG/VG protocols is selected by:

(28) $$PSS_{\Theta }^{FD,\omega }=\min\left\{\Theta_{1}^{FD}\right\}+\min\left\{\Theta_{2}^{FD,\omega }\right\},$$

where $\Theta_{1}^{\Omega }$ is given by (17) and $\Theta_{2}^{\Omega ,\omega }$ is given by (19)–(24) for $\Omega =\left\{HD,FD\right\}$ and $\omega =\left\{DF,FG,VG\right\}$, in pairs and respectively.

Since HD protocol is not impacted by the LI channel, $PSS_{\Theta }^{HD,\omega }<PSS_{\Theta }^{FD,\omega }$ is therefore obvious. The study proposes an outage threshold denoted by $T_{\Theta }$. The mechanism switches between HD and FD protocols as follows:

(29) $$PSS_{\Theta }=PSS_{\Theta }^{HD,\omega },$$

or

(30) $$PSS_{\Theta }=PSS_{\Theta }^{FD,\omega },$$

where (29) is for $T_{\Theta }<PSS_{\Theta }^{HD,\omega }<PSS_{\Theta }^{FD,\omega }$, (30) is for $PSS_{\Theta }^{HD,\omega }\approx PSS_{\Theta }^{FD,\omega }<T_{\Theta }$ and $T_{\Theta }$ is the outage threshold.

**Proposition** **2.** *The outstanding feature of NOMA is that all users are served in the same time slot by sharing the same power domain to improve the user throughput. In this research, the mechanism switches the protocols to optimize the throughput of the cooperative NOMA system. The system performance is directly proportional to the system throughput. With a higher throughput, users can reach a higher data bit rate. The throughput results of all six scenarios of $U_{1}$ and $U_{2}$ were evaluated, and the choice of the best protocol to reach the optimal system throughput was determined as:*

(31) $$PSS_{P}^{}=\max\left\{P_{1}^{\Omega }\right\}+\max\left\{P_{2}^{\Omega ,\omega }\right\}=\max\left\{\left(1-\Theta_{1}^{\Omega }\right)R_{1}^{*}\right\}+\max\left\{\left(1-\Theta_{2}^{\Omega ,\omega }\right)R_{2}^{*}\right\}.$$

**Proposition** **3.** *Given the battery capacity limitations, G-WN technology requires as little as possible energy to be spent. In this study, the EE results of all six scenarios were investigated and presented. In these results, the mechanism selects the best EE protocol for bits of data per joule (b/J) transmitted through the network considered by the $PSS_{EE}$ as:*

(32) $$PSS_{EE}^{}=\max\left\{EE_{}^{\Omega ,\omega }\right\}=\frac{\max\left\{P_{1}^{\Omega }\right\}+\max\left\{P_{2}^{\Omega ,\omega }\right\}}{\rho_{0}+\rho_{1}}.$$

## 4. Numerical Results and Discussion

The results presented below are true and accurate to the best of our knowledge without any copying from any previous research results. This article uses the following simulation parameters as Table 1:

**Note**: In all figures, the markers indicate the analysis results while the solid or dashed lines indicate the Monte Carlo simulation results. The simulation results are based on the statistics of $10^{6}$ samples. Monte Carlo simulation results are used to compare and verify the analysis results. Where they are approximated together, the analysis results can be accepted. Certain previous studies included no simulation result. In this study, we propose an algorithm for Monte Carlo simulation to investigate the outage probability as Algorithm 1:

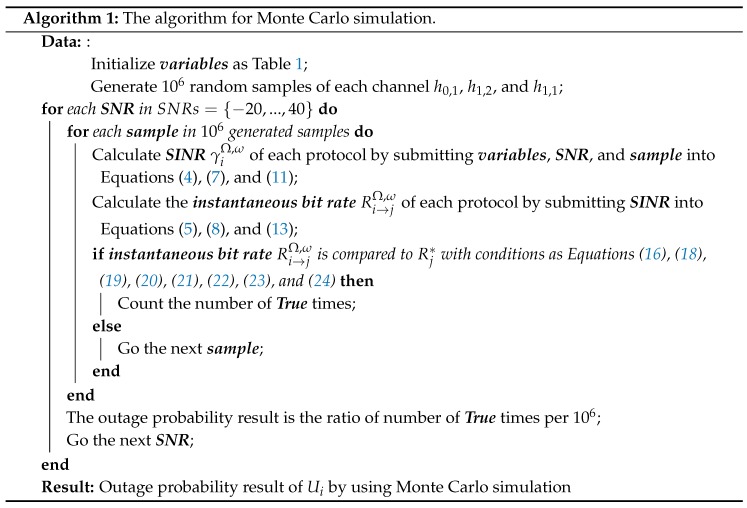



### 4.1. Numerical Results and Discussion for Outage Probability

**Figure 3:**
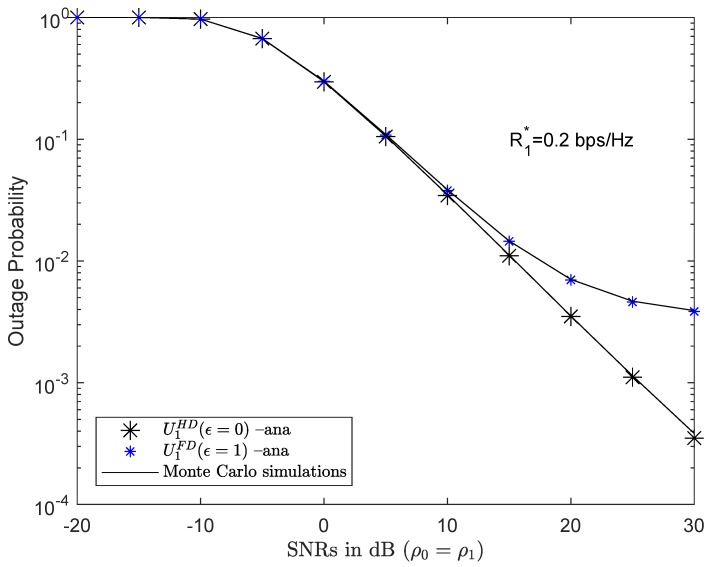
Outage probability of $U_{1}$ in HD/FD mode.

The results of the first analysis enable evaluating the outage probability at $U_{1}$ in both HD and FD mode. With the same simulation parameters as in Table 1, the HD scenario yielded better outage probability results than the FD scenario. For low SNRs, the outage probability results of $U_{1}$ in HD and FD mode were approximately the same, as for instance where SNR was 0 dB. However, as SNR increased, the results of the $U_{1}$’s outage probability in the FD mode worsened compared to the HD mode since in the FD scenario, $U_{1}$ was affected by the LI channel from its own transmitter antenna to the receiver antenna. The impact of the LI channel became more and more powerful and affected the QoS of $U_{1}$. However, the analysis results of $U_{1}$ still obtained good results, as shown in Figure 3.

To ensure fairness for both $U_{1}$ and $U_{2}$, the instantaneous bit rate thresholds that must be achieved in both $U_{1}$ and $U_{2}$ for $R_{1}^{*}=R_{2}^{*}=0.2$ bps/Hz were selected.

**Figure 4:**
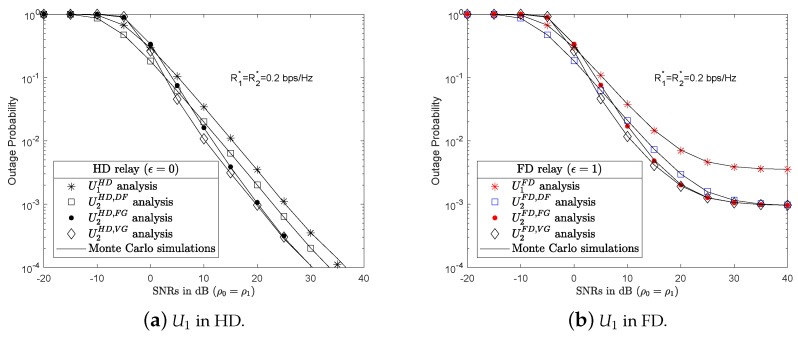
Outage probability of $U_{1}$ and $U_{2}$ in HD/FD and DF/AF with FG/VG protocols.

Next, the outage probability of $U_{2}$ with $U_{1}$ in HD/FD mode and AF with FG/VG protocols was investigated. Figure 4a,b show the outage probability results of $U_{2}$ with $U_{1}$ in HD and FD relaying mode, respectively.

Figure 4a shows $U_{1}$ in the HD mode. At SNRs lower than 0 dB, $U_{2}$ cooperated with $U_{1}$ in HD and DF protocols, the results of which are presented as square markers, yielded a better outage probability result than the other protocols. The advantage of the DF protocol is its simplicity, and $U_{2}$ only has to decode its own information $x_{2}$. Meanwhile $U_{1}$ works in the AF protocol with FG/VG, and $U_{2}$ decodes its own information $x_{2}$ symbol with the impact of noise $x_{1}$ and AWGNs $n_{1}$ and $n_{2}$. As SNRs increased, the outage probability results in the AF with FG/VG protocols improved and surpassed those with the DF protocol. At low SNRs, the outage probability results of $U_{2}$ in AF with FG and AF with VG were approximately the same. As SNRs increased, AF with VG protocol surpassed AF with the FG protocol, for example at SNRs at 5 dB. However, with higher SNRs, the AF with both FG/VG converged and were better than the DF protocol. Furthermore, the outage probability results of $U_{2}$ in all the proposed scenarios with the cooperation of $U_{1}$ were better than without any relay support. These results show the effectiveness of cooperative multi-access wireless communication over the channel fading. The results also show that no protocol outperforms any other protocol. The aim of this study was to propose a protocol switching mechanism.

Figure 4b shows $U_{1}$ in the FD mode. In the DF scenario, $U_{1}$ forwards a signal as Equation (6) to $U_{2}$. However, $U_{1}$ is affected by the LI channel $h_{1,1}$ from its own transmission antenna, thereby influencing the result of $U_{2}$’s outage probability. $U_{2}$ achieved a better outage probability result $U_{1}$, as shown in Figure 4a,b, because of $U_{1}$’s cooperation and prioritized allocation of greater power factors. As a consensus, the FD and DF scenario also have outage probability results better than FD and AF with FG/VG at low SNRs, for example, at SNRs $\rho_{0}=\rho_{1}=-5$ dB. However, as SNRs increased, the results of the outage probability of $U_{2}$ in AF with both FG and VG protocols improved and were better than the DF protocol. The AF protocol with VG yielded better results than the AF protocol with FG at some SNRs, for example at SNRs $\rho_{0}=\rho_{1}=\left\{-5,\ldots ,20\right\}$ dB. The AF protocol with VG was better than the AF protocol with FG in both Figure 4a,b. As SNRs increased, the outage probability results of both AF with VG and AF with FG scenarios reached approximately the same results, but better than in the case of the DF protocol.

### 4.2. Numerical Results and Discussion for System Throughput

**Figure 5:**
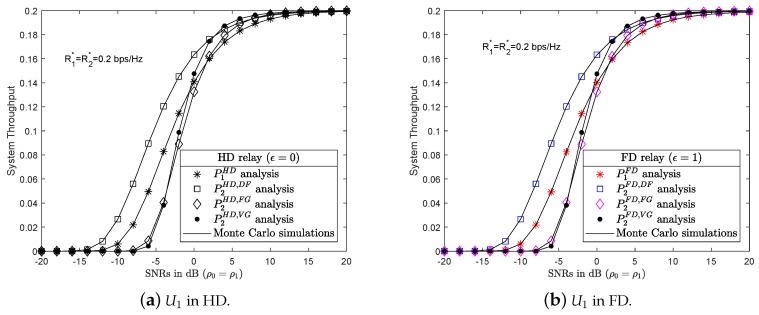
System throughput of $U_{2}$ in HD/FD and DF/AF with FG and VG protocols.

The achievable system throughput of all six scenarios is examined in this section. Figure 5a,b show that the DF protocol provide better throughput results than the other protocols in almost all SNRs. As the SNRs increased, the achievable throughput results of $U_{2}$ in both AF with FG and VG scenarios improved. However, an interesting observation can be made in Figure 5a,b. At some SNRs, AF with VG protocol returns better throughput results than the DF protocol, for example at SNRs $\rho_{0}=\rho_{1}=5$ dB. Finally, as SNRs keep increasing, the achievable throughput of all scenarios approximate and reach the threshold $R_{2}^{*}=0,2$ bps/Hz.

### 4.3. Numerical Results and Discussion for Energy Efficiency

**Figure 6:**
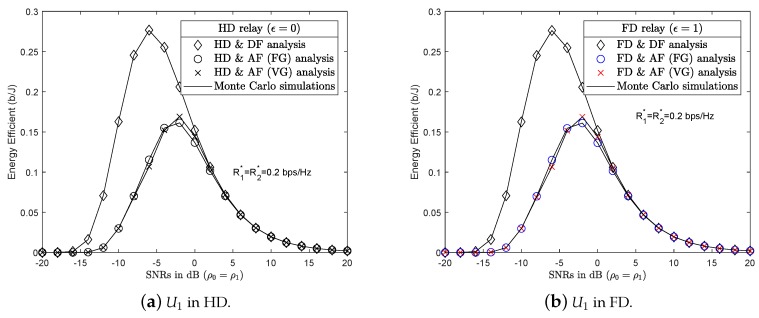
EE of HD/FD and DF, HD/FD and AF with FG/VG scenarios.

Energy waste is a serious problem that affects the living environment. G-WNs are being studied by researchers for their environmentally friendly potential. In a G-WNs network, devices must consume the least amount of energy for the total amount of data transferred and still ensure QoS for users. This section describes the deployment of EE in wireless communications. Figure 6a,b show the impact of the incorporated EE in all six scenarios, with HD and FD Relay, respectively. At almost all SNRs, the DF protocol returned superior EE results than the AF protocol with FG/VG since the DF protocol used 100% power for forwarding the $x_{2}$ symbol without including noise, as with the AF protocol. As in previous studies, the authors often assumed that the power of the BS and relay were equal to simplify their simulations. This study, however, investigates the differing transmission powers of the BS and relay to find the optimal transmission power of the relay corresponding to the power of BS of the next section.

### 4.4. Protocol Switching Mechanism

In Section 3.4, a protocol switching mechanism was designed for three proposed PSSs. In this section, these PSSs will be applied in order to ascertain the best protocol.

#### 4.4.1. PSS Based on Outage Probability

**Figure 7:**
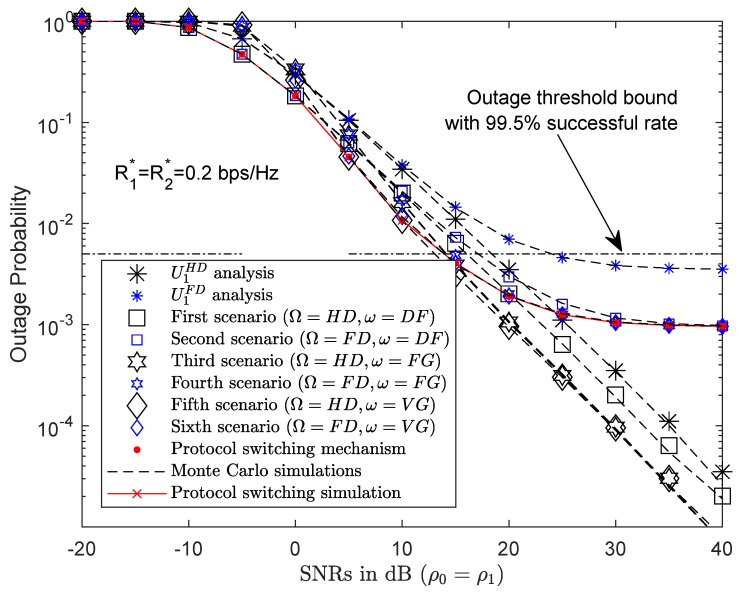
Outage probability results of PSS.

As shown in Figure 4a,b, the results of outage probability in both $U_{1}$ and $U_{2}$ effectively depend on the protocols used at $U_{1}$ corresponding to different SNRs. Therefore, the protocols were switched to ensure the best system performance as described in Proposition 1. Before the signal is forwarded to the next user, the relay pre-evaluates system performance and selects the best protocol. Figure 7 shows the outage probability results of all six scenarios. Table 2 presents the outage probability of each scenario and protocol, the minimum value (in bold font) being the best at the same SNR. The outage threshold $T_{\Theta }=0.005$. Meanwhile, the signal was forwarded from $U_{1}$ to $U_{2}$ with a 99.5% rate of success. At low SNRs, the outage probability result of $U_{2}$ with HD/FD and DF protocols at the relay is approximately the same, but better than other protocols at, for example, SNR $\rho_{0}=\rho_{1}=-5$ dB. The simulation results were generated in Matlab simulation software and are shown in Table 2. The simulation results show that the system performance depends on the transition protocol at $U_{1}$ and the SNRs. A PSS is therefore necessary in order to select the appropriate protocol for optimal system performance. By applying (27), the PSS performed system performance evaluations in all six scenarios to select the optimal protocol for user service quality, as shown in Figure 7. Furthermore, the PSS’s outage results achieved the expected outage threshold $T_{\Theta }=0.005$ in SNRs greater than 10 dB. SS’s outage results did not improve with increasing SNR, and were parallel with the horizon, demonstrating that, despite the increasing capacity, the system performance shall not improve. Moreover, energy is wasted.

#### 4.4.2. PSS Based on Throughput

**Figure 8:**
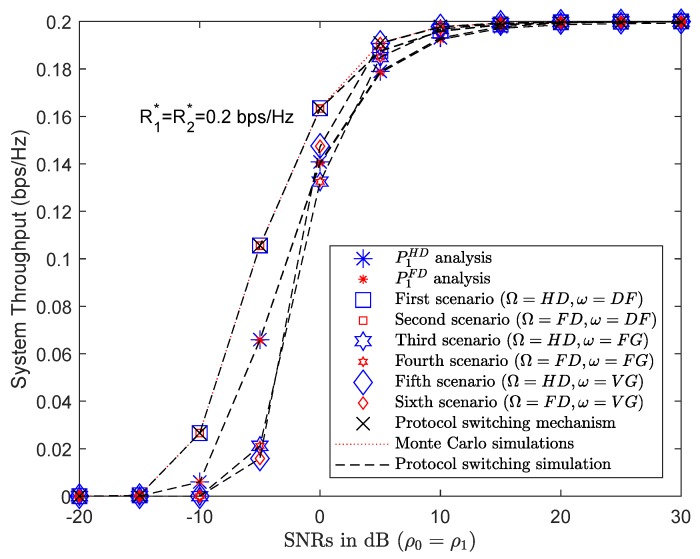
System throughput results of PSS.

Figure 8 shows a comparison of the throughput achieved at $U_{2}$ in all six scenarios. The results of the analysis and simulations of all six scenarios were extracted correctly from Matlab software and presented in Table 3. The achieved throughput of all scenarios with HD or FD approximate each other, with the HD and FD protocol markers overlapping. By applying (31), the PSS evaluates the system throughput in all six scenarios in order to select the protocol with the highest system throughput, as indicated by the red-dotted line in Figure 8. At SNR = 5 dB, it can be seen that PSS selects the AF protocol with VG instead of the DF protocol.

#### 4.4.3. PSS Based on EE

**Figure 9:**
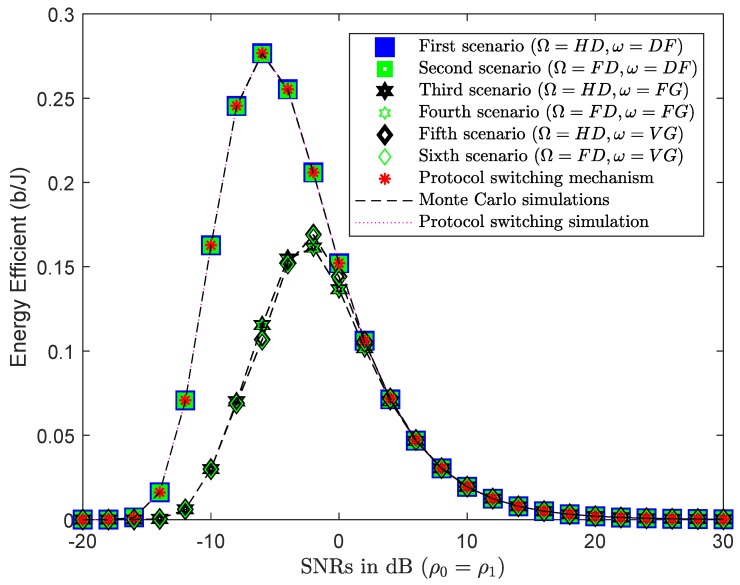
EE results of PSS.

Figure 9 compares the results of EE in all six scenarios. Although $U_{1}$ was operated in HD or FD mode, the energy efficiency of these scenarios were approximately the same. The scenarios of HD/FD and DF, in pairs, had more EE results than the AF scenario with FG/VG. In the HD/FD and DF scenarios, the received signal at $U_{2}$ only had the information symbol $x_{2}$ as (6) without sharing the transmission power factor with symbol $x_{1}$ as (10). The DF protocol therefore reached higher throughput and better EE than the AF protocol at low SNRs. However, EE in all six scenarios remained approximately the same as SNRs increased.

**Figure 10:**
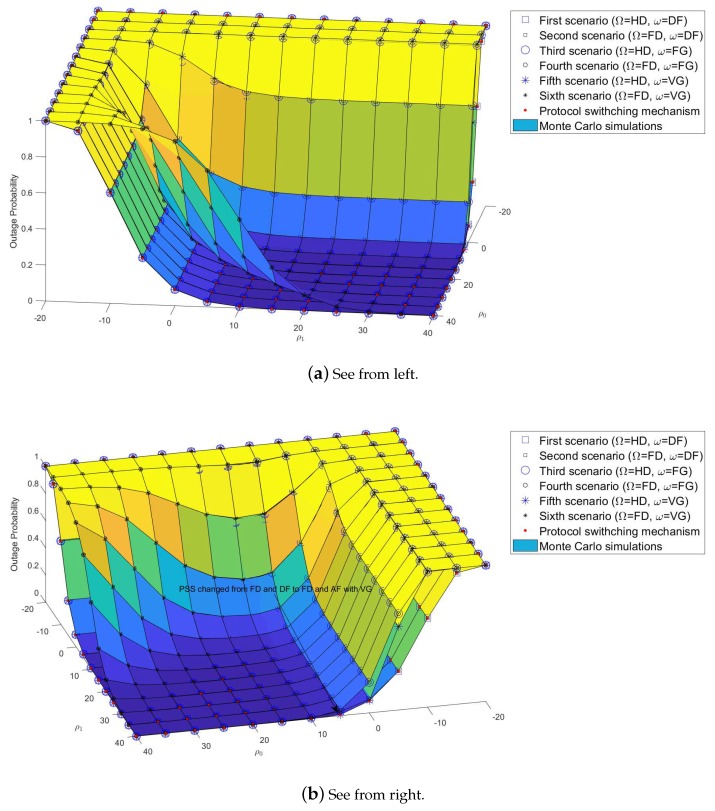
Impact of SNRs with *ρ*_0_ = {−20, ..., 40} and *ρ*_1_ = {−20, ..., 40}.

As shown in Figure 3, Figure 4, Figure 5, Figure 6, Figure 7, Figure 8 and Figure 9, system performance was not only affected by the protocols but also the SNRs. In this section, the impact of SNRs on the system performance was researched. Instead of assuming $\rho_{0}=\rho_{1}$ as in previous investigations, SNRs that could be changed were evaluated. The objective of this investigation was to find the minimum pair of SNRs able to ensure the system performance. Figure 10a,b show the outage probability results of $U_{2}$ with vector $\rho_{0}=\left\{-20,\ldots ,40\right\}$ and vector $\rho_{1}=\left\{-20,\ldots ,40\right\}$. For example, at SNR $\rho_{0}=-20$ dB, there is no value of $\rho_{1}$ to ensure the system performance. Therefore, the BS must increase the $\rho_{0}$. For example, at $\rho_{0}=10$ dB, the PSS assigned the value $\rho_{1}=10$ dB as an optimal pairing value. Had the system continued to increase $\rho_{0}$ or $\rho_{1}$, the extra SNRs would have made the system performance decrease or be wasted, for example $\rho_{0}=10$ dB and $\rho_{1}=40$ dB, or $\rho_{0}=40$ dB and $\rho_{1}=40$ dB.

**Figure 11:**
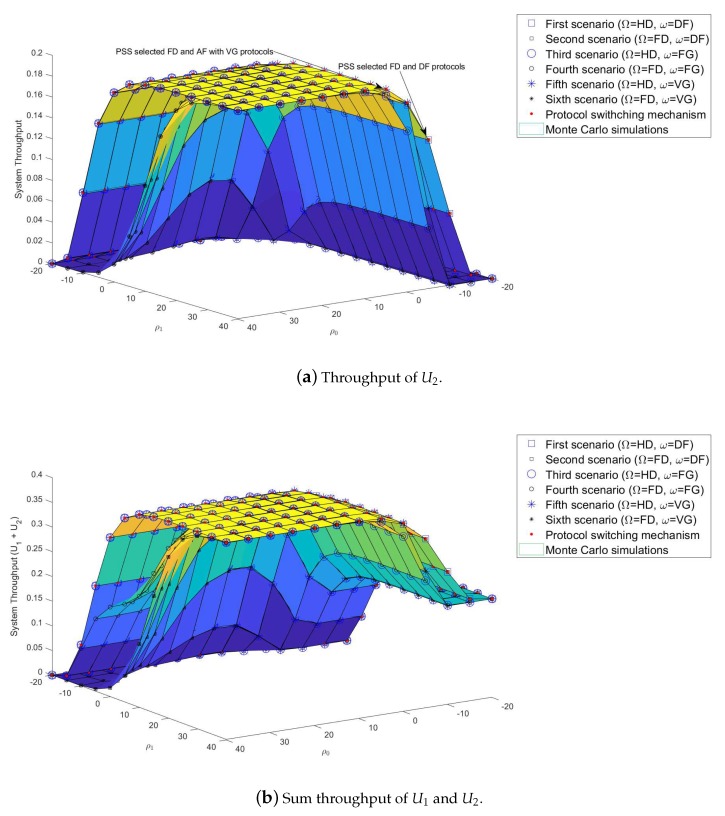
System throughput of $U_{2}$ where *ρ*_0_ = {−20, ..., 40} and *ρ*_1_ = {−20, ..., 40}.

This study also examined the impact of vector $\rho_{1}$ and vector $\rho_{2}$ on the throughput of $U_{1}$ and $U_{2}$ in the six proposed scenarios as shown in Figure 11a,b. The results of examination were compared and the best protocol selected. For example, for $\rho_{0}=40$ dB and $\rho_{1}=0$ dB, the throughput of $U_{2}$ with $U_{1}$ in the HD mode was better than others. In another example, for $\rho_{0}=0$ dB and $\rho_{1}=40$ dB, the throughput results of $U_{2}$ with $U_{1}$ in HD/FD and AF with VG protocols were better than others.

**Figure 12:**
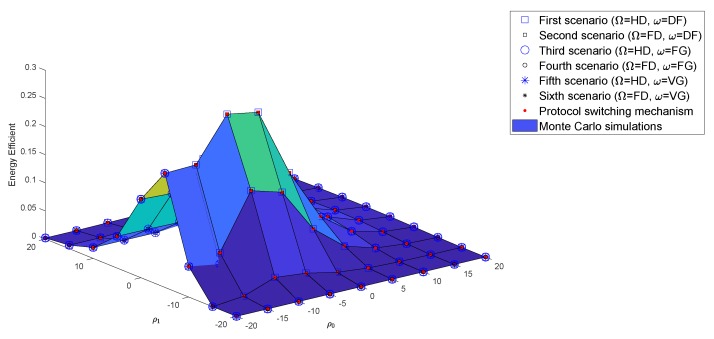
EE results for $\rho_{0}=\left\{-20,\ldots ,40\right\}$ and $\rho_{1}=\left\{-20,\ldots ,40\right\}$.

Finally, EE of the six scenarios was evaluated for $\rho_{0}=\left\{-20,\ldots ,40\right\}$ and $\rho_{1}=\left\{-20,\ldots ,40\right\}$ as shown in Figure 12. The system had the best EE with $U_{1}$ in HD/FD and DF protocols, especially at SNRs $\rho_{0}=\rho_{1}=-5$ dB, as shown in Figure 9.

## 5. Conclusions

In this study, six relay scenarios deployed in a cooperative NOMA system were examined. The results of examination indicate that no protocol was more appropriate than the others. At some SNRs, the DF protocol was more appropriate than the AF protocol, in particular at low SNRs. At other SNRs, the AF protocol was more appropriate than the DF protocol, in particular at high SNRs. This study therefore proposed a protocol switching mechanism to ascertain the optimal protocol for forwarding a signal to the next user in order to optimize system performance in terms of outage probability, system throughput and energy efficiency. A Monte Carlo simulation algorithm has also been proposed. The simulation results were used to verify with the analysis results presented in a closed form. These results of the analysis can be deployed for future G-WNs.

## Figures and Tables

**Table 1:** Simulation parameters.

Symbols	Values	Description
$h_{0,1}$	5	Channel coefficient from BS to $U_{1}$
$h_{1,2}$	3	Channel coefficient from $U_{1}$ to $U_{2}$
$h_{1,1}$	0.01	Loop interference channels at $U_{1}$
$\sigma_{0,1}^{2}$	5	Mean of channel from BS to $U_{1}$
$\sigma_{1,2}^{2}$	3	Mean of channel from $U_{1}$ to $U_{2}$
$\sigma_{1,1}^{2}$	0.01	Mean of loop interference channel from $U_{1}$ to $U_{2}$
$\alpha_{1}$	0.25	Allocation power factor of $U_{1}$
$\alpha_{2}$	0.75	Allocation power factor of $U_{2}$
$R_{1}^{*}$	0.2	Bit rate threshold of $U_{1}$
$R_{2}^{*}$	0.2	Bit rate threshold of $U_{2}$
$\rho_{0}$	$\left\{-20,40\right\}$	SNRs at BS (optional)
$\rho_{1}$	$\left\{-20,40\right\}$	SNRs at $U_{1}$ (optional)

**Note**: This paper uses the Monte Carlo simulation method with $10^{6}$ random samples of each channel.

**Table 2:** Comparison of the outage probability results.

Protocols	–5 dB	0 dB	5 dB	10 dB	30 dB
HD and DF	**0.471831**	**0.182791**	0.061839	0.019983	0.000201
FD and DF	0.472334	0.183569	0.062732	0.020917	0.001154
HD and AF with FG	0.894059	0.337188	0.075005	0.016100	9.6 × 10${}^{-5}$
FD and AF with FG	0.894160	0.337820	0.075887	0.017037	0.001049
HD and AF with VG	0.920599	0.262177	**0.045436**	**0.010755**	9.6 × 10${}^{-5}$
FD and AF with VG	0.920675	0.262880	0.046345	0.011697	**0.001048**
PSS	0.471831	0.182791	0.045436	0.010755	0.001048

**Note**: These statistical results were extracted from Matlab simulation software. The bold results are better than other results. Therefore, the corresponding protocols are selected by PSS.

**Table 3:** Comparison of throughput results.

Protocols	–5 dB	0 dB	5 dB	10 dB	30 dB
HD and DF	**0.105633**	**0.163441**	0.187632	0.196003	0.199959
FD and DF	0.105533	0.163286	0.187453	0.195816	0.199769
HD and AF with FG	0.021188	0.132562	0.184998	0.196779	0.199980
FD and AF with FG	0.021167	0.132435	0.184822	0.196592	0.199790
HD and AF with VG	0.015880	0.147564	**0.190912**	**0.197848**	0.199980
FD and AF with VG	0.015864	0.147423	0.190730	0.197660	**0.199790**
PSS	0.105633	0.163441	0.190912	0.197848	0.199790

**Note**: This paper uses the Monte Carlo simulation method with $10^{6}$ iterations. The bold results are better than other results. Therefore, the corresponding protocols are selected by PSS.

## Abbreviations

The following abbreviations are used in this manuscript:

**No.**	**Abbreviations**	**Full Description**
1	AF	Amplify-and-forward
2	AWGNs	Additive white Gaussian noises
3	BS	Base station
4	CDF	Cumulative distribution function
5	CSI	Channel state information
6	DF	Decode-and-forward
7	EE	Energy efficient
8	FD	Full-duplex
9	Fig.	Figure
10	FG	Fixed gain
11	G-WNs	Green-wireless networks
12	HD	Half-duplex
13	NOMA	Non-orthogonal multiple access
14	PDF	Probability density function
15	PSM	Protocol switching mechanism
16	PSS	Protocol switching selection
17	QoS	Quality of service
18	S	Source
19	SIC	Successive interference cancellation
20	SINR	Signal-to-interference-plus-noise ratio
21	SNR	Signal-to-noise ratio
22	$U_{i}$	The *i*-th user
23	VG	Variable gain

## Appendix A

**Proof** **of** **Theorem** **1.** This section presents the outage probability of $U_{1}$ in HD/FD mode. By applying (3) and (4) into (5) and combining with the conditional outage in (16), we obtain the following expression:

(A1) $$\begin{array}{c} {\Theta_{1}}^{\Omega }=1-\prod_{j=1}^{2}\mathrm{Pr}\left(R_{1\to j}^{\Omega }>R_{j}^{*}\right)=1-\mathrm{Pr}\left(R_{1\to 1}^{\Omega }>R_{1}^{*},R_{1\to 2}^{\Omega }>R_{2}^{*}\right)\\ =1-\mathrm{Pr}\left(\left\{\underset{\lambda_{0}}{\underset{︸}{{\left|h_{0,1}\right|}^{2}>\frac{R_{1}^{**}\left(\varepsilon {\left|h_{1,1}\right|}^{2}\rho_{1}+1\right)}{\alpha_{1}\rho_{0}},{\left|h_{1,1}\right|}^{2}>0}}\right\},\left\{\underset{\lambda_{1}}{\underset{︸}{{\left|h_{0,1}\right|}^{2}>\frac{R_{2}^{**}\left(\varepsilon {\left|h_{1,1}\right|}^{2}\rho_{1}+1\right)}{\left(\alpha_{2}-\alpha_{1}R_{2}^{**}\right)\rho_{0}},{\left|h_{1,1}\right|}^{2}>0}}\right\}\right). \end{array}$$

The expression $\lambda_{0}$ in (A1) can be solved by applying PDF (14), as follows:

(A2) $$\lambda_{0}=\int_{0}^{\infty }\int_{\frac{R_{1}^{**}\left(\varepsilon y\rho_{1}+1\right)}{\alpha_{1}\rho_{0}}}^{\infty }\frac{1}{\sigma_{0,1}^{2}\sigma_{1,1}^{2}}e^{-\left(\frac{x}{\sigma_{0,1}^{2}}+\frac{y}{\sigma_{1,1}^{2}}\right)}dxdy=e^{-\frac{R_{1}^{**}}{\alpha_{1}\rho_{0}\sigma_{0,1}^{2}}}\frac{\alpha_{1}\rho_{0}\sigma_{0,1}^{2}}{\alpha_{1}\rho_{0}\sigma_{0,1}^{2}+\varepsilon R_{1}^{**}\rho_{1}\sigma_{1,1}^{2}}.$$

Similarly, $\lambda_{1}$ in (A1) is also solved and presented as

(A3) $$\lambda_{1}=\int_{0}^{\infty }\int_{\frac{R_{2}^{**}\left(\varepsilon y\rho_{1}+1\right)}{\left(\alpha_{2}-\alpha_{1}R_{2}^{**}\right)\rho_{0}}}^{\infty }\frac{1}{\sigma_{0,1}^{2}\sigma_{1,1}^{2}}e^{-\left(\frac{x}{\sigma_{0,1}^{2}}+\frac{y}{\sigma_{1,1}^{2}}\right)}dxdy=e^{-\frac{R_{2}^{**}}{\left(\alpha_{2}-\alpha_{1}R_{2}^{**}\right)\rho_{0}\sigma_{0,1}^{2}}}\frac{\left(\alpha_{2}-\alpha_{1}R_{2}^{**}\right)\rho_{0}\sigma_{0,1}^{2}}{\left(\alpha_{2}-\alpha_{1}R_{2}^{**}\right)\rho_{0}\sigma_{0,1}^{2}+\varepsilon R_{2}^{**}\rho_{1}\sigma_{1,1}^{2}}.$$

where ε=0, then $U_{1}$ operates in the HD mode; and where ε=1, then $U_{1}$ operates in the FD mode. □

**Proof** **of** **Remarks** **1** **and** **2.** This section presents the outage probability of $U_{2}$ with $U_{1}$ in HD/FD and DF protocols. By applying (7) into (8) and combining with the conditional outage in (19) or (20), we obtain the following expression:

(A4) $$\begin{array}{cc} {\Theta_{2}}^{\Omega ,DF} & =1-\prod_{i=1}^{2}\mathrm{Pr}\left(R_{i\to 2}^{\Omega ,DF}>R_{2}^{*}\right)=1-\mathrm{Pr}\left(R_{1\to 2}^{\Omega }>R_{2}^{*},R_{1\to 2}^{\Omega ,DF}>R_{2}^{*}\right)\\ & =1-\mathrm{Pr}\left(\left\{\underset{\lambda_{1}}{\underset{︸}{{\left|h_{0,1}\right|}^{2}>\frac{R_{2}^{**}\left(\varepsilon {\left|h_{1,1}\right|}^{2}\rho_{1}+1\right)}{\left(\alpha_{2}-\alpha_{1}R_{2}^{**}\right)\rho_{0}},{\left|h_{1,1}\right|}^{2}>0}}\right\},\left\{\underset{\lambda_{2}}{\underset{︸}{{\left|h_{1,2}\right|}^{2}>\frac{R_{2}^{**}}{\rho_{1}}}}\right\}\right), \end{array}$$

where $\Omega =\left\{HD,FD\right\}$ and $\lambda_{1}$ in (A4) is given by (A3). It is not necessary to rewrite. $\lambda_{2}$ can be solved by applying the PDF (14) as follows:

(A5) $$\lambda_{2}=\int_{\frac{R_{2}^{**}}{\rho_{1}}}^{\infty }\frac{1}{\sigma_{1,2}^{2}}e^{-\frac{x}{\sigma_{1,2}^{2}}}dx=e^{-\frac{R_{2}^{**}}{\rho_{1}\sigma_{1,2}^{2}}}.$$

The important advantage of the DF protocol over the AF protocol is the simplicity of both calculation and simulation. □

**Proof** **of** **Remark** **3** **and** **4.** This section presents the outage probability of $U_{2}$ with $U_{1}$ in HD/FD and AF with FG protocols. By submitting (11) for ω=FG into (13) and combining with the conditional outage in (21) or (22), we obtain the following expression:

(A6) $${\Theta_{2}}^{\Omega ,FG}=1-\prod_{i=1}^{2}\mathrm{Pr}\left(R_{i\to 2}^{\Omega ,FG}>R_{2}^{*}\right)=1-\mathrm{Pr}\left(\underset{\lambda_{1}}{\underset{︸}{R_{1\to 2}^{\Omega }>R_{2}^{*}}},\underset{\lambda_{3}}{\underset{︸}{R_{1\to 2}^{\Omega ,FG}>R_{2}^{*}}}\right),$$

where the $\lambda_{1}$ in (A6) is still given by (A3), and $\lambda_{3}$ can be solved as follows:

(A7) $$\begin{array}{cc} \lambda_{3} & =\mathrm{Pr}\left({\left|h_{0,1}\right|}^{2}>\frac{R_{2}^{**}\left(\varepsilon {\left|h_{1,1}\right|}^{2}\rho_{1}+1+\frac{\rho_{0}\sigma_{1,2}^{2}+1}{\rho_{1}^{2}{\left|h_{1,2}\right|}^{2}}\right)}{\left(\alpha_{2}-\alpha_{1}R_{2}^{**}\right)\rho_{0}},{\left|h_{1,2}\right|}^{2}>0,{\left|h_{1,1}\right|}^{2}>0\right)\\ & =\int_{0}^{\infty }\int_{0}^{\infty }\int_{\frac{R_{2}^{**}\left(\varepsilon z\rho_{1}+1+\frac{\rho_{0}\sigma_{1,2}^{2}+1}{\rho_{1}^{2}y}\right)}{\left(\alpha_{2}-\alpha_{1}R_{2}^{**}\right)\rho_{0}}}^{\infty }\frac{1}{\sigma_{0,1}^{2}\sigma_{1,2}^{2}\sigma_{1,1}^{2}}e^{-\left(\frac{x}{\sigma_{0,1}^{2}}+\frac{y}{\sigma_{1,2}^{2}}+\frac{z}{\sigma_{1,1}^{2}}\right)}dxdydz\\ & =\frac{e^{-\frac{R_{2}^{**}}{\left(\alpha_{2}-\alpha_{1}R_{2}^{**}\right)\rho_{0}\sigma_{0,1}^{2}}}2\sqrt{\frac{1}{\sigma_{1,2}^{2}}}\left(\alpha_{2}-\alpha_{1}R_{2}^{**}\right)\rho_{0}\sigma_{0,1}^{2}K_{1}\left(\frac{2\sqrt{\frac{1}{\sigma_{1,2}^{2}}}}{\sqrt{\frac{\left(\alpha_{2}-\alpha_{1}R_{2}^{**}\right)\rho_{0}\rho_{1}^{2}\sigma_{0,1}^{2}}{R_{2}^{**}\left(1+\rho_{0}\sigma_{1,2}^{2}\right)}}}\right)}{\left(\left(\alpha_{2}-\alpha_{1}R_{2}^{**}\right)\rho_{0}\sigma_{0,1}^{2}+\varepsilon R_{2}^{**}\rho_{1}\sigma_{1,1}^{2}\right)\sqrt{\frac{\left(\alpha_{2}-\alpha_{1}R_{2}^{**}\right)\rho_{0}\rho_{1}^{2}\sigma_{0,1}^{2}}{R_{2}^{**}\left(1+\rho_{0}\sigma_{1,2}^{2}\right)}}}. \end{array}$$

where ϵ=0 in both $\lambda_{1}$ and $\lambda_{3}$, then $U_{1}$ operates in HD and AF with FG protocols. Where ϵ=1 in both $\lambda_{1}$ and $\lambda_{3}$, then $U_{1}$ operates in FD and AF with FG protocols. □

**Proof** **of** **Remarks** **5** **and** **6.** This section presents the outage probability of $U_{2}$ with $U_{1}$ in HD/FD and AF with VG protocols. By submitting (11) for ω=VG into (13) and combining with the conditional outage in (23) or (24), we obtain the following expression:

(A8) $${\Theta_{2}}^{\Omega ,VG}=1-\prod_{i=1}^{2}\mathrm{Pr}\left(R_{i\to 2}^{\Omega ,FG}>R_{2}^{*}\right)=1-\mathrm{Pr}\left(\underset{\lambda_{1}}{\underset{︸}{R_{1\to 2}^{\Omega }>R_{2}^{*}}},\underset{\lambda_{4}}{\underset{︸}{R_{1\to 2}^{\Omega ,VG}>R_{2}^{*}}}\right),$$

where $\lambda_{1}$ is also given by (A3), and $\lambda_{4}$ can be solved as follows:

(A9) $$\begin{array}{cc} \lambda_{4} & =\mathrm{Pr}\left({\left|h_{0,1}\right|}^{2}>\frac{R_{2}^{**}\left(\varepsilon {\left|h_{1,1}\right|}^{2}\rho_{1}+1+\frac{\rho_{0}{\left|h_{1,2}\right|}^{2}+1}{\rho_{1}^{2}{\left|h_{1,2}\right|}^{2}}\right)}{\left(\alpha_{2}-\alpha_{1}R_{2}^{**}\right)\rho_{0}},{\left|h_{1,2}\right|}^{2}>0,{\left|h_{1,1}\right|}^{2}>0\right)\\ & =\int_{0}^{\infty }\int_{0}^{\infty }\int_{\frac{R_{2}^{**}\left(\varepsilon z\rho_{1}+1+\frac{\rho_{0}y+1}{\rho_{1}^{2}y}\right)}{\left(\alpha_{2}-\alpha_{1}R_{2}^{**}\right)\rho_{0}}}^{\infty }\frac{1}{\sigma_{0,1}^{2}\sigma_{1,2}^{2}\sigma_{1,1}^{2}}e^{-\left(\frac{x}{\sigma_{0,1}^{2}}+\frac{y}{\sigma_{1,2}^{2}}+\frac{z}{\sigma_{1,1}^{2}}\right)}dxdydz\\ & =\frac{e^{-\frac{R_{2}^{**}\left(\rho_{1}^{2}+\rho_{0}\right)}{\left(\alpha_{2}-\alpha_{1}R_{2}^{**}\right)\rho_{0}\rho_{1}^{2}\sigma_{0,1}^{2}}}2\sqrt{\frac{1}{\sigma_{1,2}^{2}}}\left(\alpha_{2}-\alpha_{1}R_{2}^{**}\right)\rho_{0}\sigma_{0,1}^{2}K_{1}\left(\frac{2\sqrt{\frac{1}{\sigma_{1,2}^{2}}}}{\sqrt{\frac{\left(\alpha_{2}-\alpha_{1}R_{2}^{**}\right)\rho_{0}\rho_{1}^{2}\sigma_{0,1}^{2}}{R_{2}^{**}}}}\right)}{\left(\left(\alpha_{2}-\alpha_{1}R_{2}^{**}\right)\rho_{0}\sigma_{0,1}^{2}+\varepsilon R_{2}^{**}\rho_{1}\sigma_{1,1}^{2}\right)\sqrt{\frac{\left(\alpha_{2}-\alpha_{1}R_{2}^{**}\right)\rho_{0}\rho_{1}^{2}\sigma_{0,1}^{2}}{R_{2}^{**}}}}. \end{array}$$

where ϵ=0 in both $\lambda_{1}$ and $\lambda_{4}$, then $U_{1}$ operates in HD and AF with VG protocols. Where ϵ=1 in both $\lambda_{1}$ and $\lambda_{4}$, then $U_{1}$ operates in FD and AF with VG protocols. End of proof. □
